# The association of genetic polymorphisms in interleukin-1 receptors type 1 and type 2 with sudden sensorineural hearing loss in a Taiwanese population: a case control study

**DOI:** 10.1186/s40463-021-00550-w

**Published:** 2021-12-05

**Authors:** Chen-Yu Chien, Shu-Yu Tai, Kuan-Hui Li, Hua-Ling Yang, Leong-Perng Chan, Edward Hsi, Ling-Feng Wang, Kuen-Yao Ho, Ning-Chia Chang

**Affiliations:** 1 Department of Otorhinolaryngology, School of Medicine, College of Medicine, Kaohsiung Medical University, Kaohsiung, Taiwan; 2 Department of Family Medicine, School of Medicine, College of Medicine, Kaohsiung Medical University, Kaohsiung, Taiwan; 3 Department of Otorhinolaryngology, Kaohsiung Medical University Hospital, Kaohsiung Medical University, No.100, Tzyou 1st Road, Kaohsiung, 807 Taiwan; 4 Family MedicineDepartment of Family Medicine, Kaohsiung Medical University Hospital, Kaohsiung Medical University, Kaohsiung, Taiwan; 5 Division of Hepatobiliary and Pancreatic Medicine, Department of Internal Medicine, Kaohsiung Medical University Hospital, Kaohsiung Medical University, Kaohsiung, Taiwan; 6 Department of Medical Research, Kaohsiung Medical University Hospital, Kaohsiung Medical University, Kaohsiung, Taiwan; 7 Department of Otorhinolaryngology, Kaohsiung Municipal Ta-Tung Hospital, Kaohsiung, Taiwan; 8 Department of Family Medicine, Kaohsiung Municipal Ta-Tung Hospital, Kaohsiung, Taiwan; 9 Department of Otorhinolaryngology, Kaohsiung Municipal Siaogang Hospital, Kaohsiung, Taiwan

**Keywords:** Sudden sensorineural hearing loss, Interleukin-1 receptor, Genetic polymorphism

## Abstract

**Background:**

Sudden sensorineural hearing loss (SSNHL) is a disease with an unknown etiology; damage to the auditory nerve from inflammation due to viral infection or vascular incidents has been implicated. According to several studies, cytokines, including interleukins, are associated with SSNHL in terms of serum expression and genetic polymorphisms. Interleukin-1 (IL-1) plays a key role in inflammation and may be associated with SSNHL. This study analyzed the association of single nucleotide polymorphisms (SNPs) of IL-1 receptor (IL-1R) genes with SSNHL in Taiwan.

**Methods:**

We conducted a case–control study involving 401 patients with SSNHL and 730 healthy controls. Four SNPs (IL-1R type 1 gene [*IL1R1*] [rs3917225 and rs2234650] and IL-1R type 2 gene [*IL1R2*] [rs4141134 and rs2071008]) were selected. The genotypes were determined using the TaqMan assay. The Hardy–Weinberg equilibrium (HWE) was tested for each SNP, and genetic effects were evaluated.

**Results:**

The TT genotype of rs2234650 had an adjusted odds ratio (OR) of 2.988 (95% confidence interval [95% CI] 1.27–6.82) (*P* = 0.012) compared with the CC genotype in patients with SSNHL. The SNP rs2234650 was associated with SSNHL in the recessive model (TT vs. CC + CT, *P* = 0.0206, OR = 2.681). The CT genotype of rs4141134 had an adjusted OR of 3.860 (95% CI 2.01–7.44; *P* < 0.0001) compared with the TT genotype, in patients with SSNHL. The SNP rs4141134 was associated with SSNHL under the dominant model (CC + CT vs. TT, *P* < 0.0001, OR = 4.087).

**Conclusion:**

These findings suggest that *IL1R1 and IL1R2* gene polymorphisms may contribute to an increased risk of SSNHL in Taiwan.

## Background

Sudden sensorineural hearing loss (SSNHL) is defined as a loss of at least 30 dB across three contiguous frequencies occurring within 3 days [1, 2]. The estimated incidence is between 5 and 20 per 100,000 persons per year in the United States [1]. In Taiwan, the incidence rates per 100,000 people are 8.85 for men and 7.79 for women [3]. Various causes of SSNHL have been postulated, including viral infection, vascular compromise, intracochlear membrane rupture, autoimmune inner ear disease, and genetic factors [4]. Damage to the auditory nerve from inflammation due to viral infection and vascular incidents has also been suggested [5]**.** However, no consensus has been reached on such causes, and the etiology and pathogenesis of SSNHL remain controversial.

SSNHL and age-related hearing loss may be associated with inflammation-related genetic factors, including interleukins, heat shock proteins, and tumor necrosis factor (TNF) [4–8]. Interleukins, which plays a central part in transfer information, activated and regulates immune cells, mediated T and B cell activation and inflammatory response, is a type of cytokine produced by multiple cells and acts on various cells [7]. Interleukin-1 (IL-1) is among the most powerful inflammatory cytokines. The IL-1 family, including agonists IL-1α and IL-1β and their antagonist IL-1 receptor antagonist (IL-1Ra), are integral components of the innate immune system.

Furuta et al. reported a significant association of both SSNHL and Ménière disease with IL-1α − 889 C/T (rs1800587) polymorphism in Japan [6]. The IL-1α − 889C⁄T polymorphism is located in the promoter region of IL-1α, and carriers of the minor T allele have been shown to exhibit increased transcriptional activity of the gene [9]. Um et al. reported a significant difference between patients with SSNHL and controls in the carriage of both the IL-1β − 511 (rs16944) T allele and the IL-1β + 3953 (rs1143627) T allele in a Korean population [5]. Their finding suggests that the IL-1β − 511 and IL-1β + 3953 loci may play crucial roles in the etiopathogenesis of SSNHL [5]. The homozygosity for the IL-1β + 3953 T allele was associated with a fourfold higher in the production of IL-1β compared to the homozygosity for the C allele [10]. The mechanism by which the IL-1β gene polymorphisms influences SSNHL is probably related to a different IL-1β synthesis, secretion, and activity.

To date, no study has examined the association of interleukin-1 receptor (IL-1R) type 1 (*IL1R1*) and type 2 (*IL1R2*) genetic polymorphisms with SSNHL. We hypothesized that the genetic polymorphisms of IL-1Rs may influence an individual’s susceptibility to SSNHL. Therefore, this study investigated the association of *IL1R1* and *IL1R2* single nucleotide polymorphisms (SNPs) with SSNHL in Taiwan.

## Methods

### Participants

We recruited 401 patients with SSNHL and 730 healthy controls from Kaohsiung Medical University Hospital, Kaohsiung, Taiwan. Patients with SSNHL were recruited according to the diagnostic criteria in the Clinical Practice Guideline: Sudden Hearing Loss from the American Academy of Otolaryngology–Head and Neck Surgery [11]. Healthy volunteers without a history of hearing loss or any hearing disorder were enrolled as controls. Baseline demographic information was collected on both groups. The SSNHL group underwent audiometric tests, including pure tone audiometry, auditory brainstem–evoked responses, and computer tomography or magnetic resonance imaging (to exclude acoustic neuroma). The pattern of the audiogram was categorized by Sheehy classification [12]. The audiograms were categorized into 4 patterns, which were low tone, high tone, flat, and total hearing loss types.

### SNP selection and genotyping

*IL1R1* and *IL1R2* genetic polymorphisms rs3917225, rs2234650 (also known as Pst-1 1970C/T), rs4141134, and rs2071008 were selected as the target SNPs, as referenced from earlier studies [13, 14]. Genomic DNA was extracted from the peripheral blood using standard methods. Genotyping was performed using the TaqMan assay (ABI 7500 Real-Time PCR System, Applied Biosystems, Foster City, CA), and reactions were performed in 96-well microplates in ABI 9700 thermal cyclers (Applied Biosystems). Fluorescence was measured using the ABI 7500 Real-Time PCR System and analyzed using SDS software version 1.2.3 (Applied Biosystems). All SNPs were typed in each participant.

### Statistical analysis

Continuous variables were analyzed using independent *t* tests, the results of which are presented as mean ± standard deviation (SD). Allele frequencies were obtained by direct gene counting. Categorical data were computed using a two-sided chi-squared test. We assessed the Hardy–Weinberg equilibrium for the controls by using the chi-squared test. The effect of the minor allele of each SNP was examined in both dominant and recessive genetic models. To assess the genetic effects, we performed multivariate logistic regression analyses to obtain the age- and sex-adjusted odds ratios (ORs) and their 95% confidence intervals (95% CIs). All statistical analyses were performed using JMP software version 10.0 and Stat View version 5.0 (SAS institute Inc., Cary, NC) for Windows. Two-tailed *P* values of < 0.05 were considered significant.

## Results

### Study participants

The baseline characteristics of the study participants are summarized in Table 1. The mean age of the 401 patients (216 men [54%] and 185 women [46%]) was 50.7 ± 14.6 years, and the mean age of the 730 controls (414 men [57%] and 316 women [43%]) was 71.9 ± 5.9 years. Overall, the SSNHL group was younger than the control group (*P* < 0.0001). The sex distribution was 1.17:1 (male to female) in the SSNHL group, and the sex characteristics were not significantly different between the two groups.

**Table 1.** Demographics of the participants

	SSNHL Case (n = 401)	Control (n = 730)	*P* value
Age (mean ± SD, y/o)	50.7 ± 14.6	71.9 ± 5.9	< 0.0001*^a^
Sex (n, %)			
Male	216 (54)	414 (57)	0.3565^b^
Female	185 (46)	316 (43)	
Vertigo (n, %)			
Yes	80 (20)	N/A	
No	321 (80)		
Audiometric pattern (n, %)			
Low	35 (8.7)	N/A	
High	48 (12.0)		
Flat	235 (58.6)		
Total	83 (20.7)		

^a^Independent *t* test

^b^Chia-squared test

*p < 0.05

Eighty of our SSNHL patients had vertigo (20%), and 321 did not have (80%). Thirty-five (8.7%) patients had low-frequency hearing loss; forty-eight (12.0%) patients had high-frequency hearing loss; 235 (58.6%) patients had flat type hearing loss; and 83 (20.7%) patients had total hearing loss configuration.

### Genetic analyses

#### Allele and genotype analysis

The genetic distribution in this study followed the Hardy–Weinberg equilibrium. The adjusted ORs were computed in our analyses of the associations of *IL1R1* and *IL1R2* genotypes with SSNHL. Table 2 summarizes the distributions of alleles and genotype frequencies of the *IL1R1* and *IL1R2* SNPs. The allelic and genotypic distributions were not significantly different between both groups for *IL1R1* rs3917225 and *IL1R2* rs2071008. However, *IL1R1* rs2234650 and *IL1R2* rs4141134 were significantly associated with SSNHL (Table 2).

**Table 2.** Association of for single nucleotide polymorphisms (SNPs) of *IL1R* gene and sudden sensorineural hearing loss (SSNHL) in overall study subjects

SNP	Overall, n (%)			
	SSNHL case	Control	*P* value^a^	Adjusted OR(95%CI)^b^, *P* value^a^
rs3917225				
Genotypes				
AA	154 (38)	270 (37)	*P* = 0.5845	1.00^c^
AG	179 (45)	348 (48)		0.875 (0.56–1.37), *P* = 0.556
GG	68 (17)	112 (15)		0.681 (0.36–1.25), *P* = 0.219
Alleles				
A	487 (61)	888 (61)	*P* = 0.9633	1.00^3^
G	315 (39)	572 (39)		0.833 (0.62–1.12), *P* = 0.229
rs2234650				
Genotypes				
CC	197 (49)	395 (54)	*P* = 0.0031*	1.00^c^
CT	161 (40)	296 (41)		1.273 (0.83–1.96), *P* = 0.269
TT	43 (11)	39 (5)		2.988 (1.27–6.82), *P* = 0.012*
Alleles				
C	555 (69)	1086 (74)	*P* = 0.0083*	1.00^c^
T	247 (31)	374 (26)		1.448 (1.05–2.00), *P* = 0.026*
rs4141134				
Genotypes				
TT	318 (79)	679 (93)	*P* < 0.0001*	1.00^c^
CT	82 (20)	51 (7)		3.860 (2.01–7.44), *P* < 0.0001*
CC	1 (1)	0 (0)		N/A
Alleles				
T	718 (90)	1409 (97)	*P* < 0.0001*	1.00^c^
C	84 (10)	51 (3)		3.990 (2.15–7.38), *P* < 0.0001*
rs2071008				
Genotypes				
GG	230 (57)	439 (60)	*P* = 0.3621	1.00^c^
GT	145 (36)	257 (35)		1.147 (0.74–1.77), *P* = 0.535
TT	26 (7)	34 (5)		2.233 (0.96–5.07), *P* = 0.063
Alleles				
G	605 (75)	1135 (78)	*P* = 0.2136	1.00^c^
T	197 (25)	325 (22)		1.316 (0.94–1.83), *P* = 0.107

^a^Chi-squared test

^b^Adjusted for age, sex (male; female)

^c^Reference group

*p < 0.05

The proportion of the T allele of *IL1R1* rs2234650 was higher in the SSNHL group than in the control group (31% vs. 26%, *P* = 0.0083), and patients with this allele had an increased risk of SSNHL (adjusted OR = 1.448; 95% CI 1.05–2.00, *P* = 0.026). The multivariate logistic regressions yielded a sex- and age-adjusted OR of 2.988 (95% CI 1.27–6.82, *P* = 0.012) for the TT genotype and 1.273 (95% CI 0.83–1.96, *P* = 0.269) for the CT genotype of *IL1R1* rs2234650, compared with the CC genotype (Table 2).

The proportion of the C allele of *IL1R2* rs4141134 was higher in the SSNHL group than in the control group (10% vs. 3%, *P* < 0.0001), and patients with this allele had an increased risk of SSNHL (adjusted OR = 3.990; 95% CI 2.15–7.38, *P* < 0.0001). The multivariate logistic regressions yielded a sex- and age-adjusted OR of 3.860 (95% CI 2.01–7.44, *P* < 0.0001) for the CT genotype of *IL1R2* rs4144134 compared with the TT genotype (Table 2).

A significant result was observed from the recessive model for *IL1R1* rs2234650, which yielded an adjusted OR of 2.681 (95% CI 1.17–5.95, *P* = 0.0206) for the TT genotype compared with the CC + CT genotypes after adjustment for age and sex (Table 3). Participants who carried the TT homozygote of *IL1R1* rs2234650 were associated with a higher risk of SSNHL compared with individuals with the CC + CT genotypes.

**Table 3.** The association between *IL1R* SNP rs2234650 under recessive model, and rs4141134 under dominant model with SSNHL

SNP	Case n (%)	Control, n (%)	Adjusted OR (95% CI)^b^, *P* value^a^
rs2234650 Recessive			
Genotypes			
CC + CT	358 (89%)	691 (95%)	1.00^c^
TT	43 (11%)	39 (5%)	2.681 (1.17–5.95), *P* = 0.0206*
rs4141134 Dominant			
Genotypes			
TT	318 (79%)	679 (93%)	1.00^c^
CC + CT	83 (21%)	51 (7%)	4.087 (2.14–7.83), *P* < 0.0001*

^a^Chi-squared test

^b^Adjusted for age, sex (male; female)

^c^Reference group

*p < 0.05

Another significant result was observed under the dominant model in *IL1R2* rs4141134 (Table 3), which yielded an adjusted OR of 4.087 (95% CI 2.14–7.83, *P* < 0.0001) for the CC + CT genotypes compared with the TT genotype after adjustment for age and sex (Table 3). Participants who carried at least one risk C allele (i.e., CC or CT genotype) of *IL1R2* rs4141134 were associated with a higher risk of SSNHL compared with those with the TT homozygote.

## Discussion

A significant association of SSNHL with polymorphisms involved in inflammation has been reported [7]. SSNHL may be associated with inflammation-related genetic factors, including interleukins (IL-1α, IL-1β, IL-4R, IL-6, IL-7R, and, IL-10), and TNF [4–7]**.** The IL-1 superfamily comprises the agonist IL-1α and IL-1β and their antagonist IL-1Ra. Among all the involved cytokines, TNF-α, IL-1β, IL-2, IL-8 and IL-12, and interferon-γ are proinflammatory cytokines, and IL-4, IL-6, and IL-10 are anti-inflammatory cytokines [15].

In this study, we confirmed the hypothesis that the genetic polymorphisms of the IL-1Rs are associated with susceptibility to SSNHL. These results suggest that the development of SSNHL is related to the inflammatory pathway and is underlain by individual genetic differences. IL-1 is a central mediator of innate immunity and inflammation. As a multifunctional proinflammatory cytokine, IL-1 plays a key role in inflammation by activating the expression of genes associated with innate and adaptive immune responses [14]. The biological activity of IL-1 is mediated by its receptors. IL-1R type 1 affects nuclear factor kappa B (NF-κB) signaling by combining with IL-1 on the cell surface and upregulating inflammation [16]. The *IL1R1* and *IL1R2* genes encode cytokine receptors for IL-1. Merchant et al. suggested that SSNHL might arise be due to the abnormal activation of cellular stress pathways involving NF-κB [17]. Because the pathologic activation of NF-κB can result in inflammatory cytokine production, IL-1 production may be closely associated with inner ear diseases [5]. Research has established that corticosteroids are potent inhibitors of NF-κB in multiple pathways [18]. This is consistent with the clinical response to steroids observed in patients with SSNHL [17]**.**

Chang et al. observed that the G allele of *IL1R1* rs3917225 conferred a decreased risk of age-related hearing impairment (ARHI) and that both the GG genotype of *IL1R1* rs3917225 in all studied hereditary models and the TT genotype of *IL1R2* rs2071008 in the studied recessive model conferred decreased risks of ARHI [14]. In addition, these authors reported that the SNPs rs2234650 of *IL1R1* and rs4141134 of *IL1R2* were not significantly associated with ARHI susceptibility. However, our study investigating the relationship between four SNPs in the *IL1R* genes and SSNHL in a Taiwanese population and revealed that *IL1R* exerted a genetic effect on the development of SSNHL. The significant result stemmed from the rs2234650 polymorphism of the *IL1R1* gene and rs4141134 polymorphism of the *IL1R2* gene, highlighting the role of the rare T allele of rs2234650 and C allele of rs4141134 on the risk of SSNHL in Taiwan. We identified no significant differences between the SSNHL and control groups at SNPs rs3917225 of the *IL1R1* gene and rs2071008 of the *IL1R2* gene. This investigation is the first to report the potential contribution of *IL1R* genetic variants to susceptibility to SSNHL.

In the study of Vasilyev et al., healthy individuals with the homozygous TT genotype of SNP rs2234650 had a low percentage of intact CD14^+^ monocytes expressing *IL1R1* on their surface [13]. However, Kassner et al. observed no significant difference in CD14^+^ monocytes in patients with SSNHL compared with healthy participants [19]. Our recessive model yielded a significant adjusted OR of 2.681 (*P* = 0.0206) for the TT genotype compared with the CC + CT genotypes of rs2234650 after adjustment for age and sex. This result revealed a tendency for individuals with the TT genotype to experience a higher incidence of SSNHL than do those carrying the C genotype (CC + CT), suggesting that the TT genotype conferred a moderately heightened SSNHL risk in these individuals. The T allele of rs2234650 seems to be a risk allele with a recessive effect on SSNHL in Taiwan. SNP rs2234650 of *IL1R1* had not been previously reported to be associated with hearing impairment.

IL-1 receptor 2 (IL1R2) is a molecular decoy that traps IL-1β and does not initiate subsequent signaling events, therefore, it suppresses inflammatory responses [14]. Valsilyev et al. reported that healthy individuals with the homozygous CC genotype of SNP rs4141134 had a lower density of IL1R2 on the surface of CD14^+^ monocytes in lipopolysaccharide-stimulated peripheral blood mononuclear cell cultures [13]. These individuals, however, had a higher percentage of cells expressing membrane-bound IL1R2 in the intact CD3^+^ T cell population [13]. From these previous findings, we postulate that individuals with the CC genotype of rs4141134 may have a lower level of membrane-bound IL1R2 for IL-1 binding, thus enhancing the effect of inflammation. Kassner et al. observed a significantly lower percentage of CD3^+^ T lymphocytes in patients with SSNHL compared with healthy individuals but no significant differences in CD14^+^ monocytes [19]. Furthermore, our dominant models yielded a significant adjusted OR of 4.087 (*P* < 0.0001) for CC + CT versus TT genotype of rs4141134 after adjustment for age and sex. This result revealed a tendency for individuals carrying the C genotype (CC + CT) to have a higher incidence of SSNHL than do those with the TT genotype, suggesting that either of the two C-containing genotypes conferred a degree of SSNHL risk in these individuals. The C allele of rs4141134 seems to be a risk allele with a dominant effect on SSNHL in Taiwan. SNP rs4141134 of *IL1R2* had not been previously reported to be associated with hearing impairment.

IL-1 is among the most powerful inflammatory cytokines. Pathak et al. reported that IL-1β was overexpressed and aberrantly regulated in corticosteroid nonresponders with autoimmune inner ear disease (AIED) [20]. The study of Um et al. indicated the involvement of proinflammatory cytokines, especially IL-1β, in cochlear damage. IL-1β is not only an important host genetic factor but also a key proinflammatory cytokine that can regulate the expression of several molecules involved in inflammation [5]. Furuta et al. reported that in a Japanese population, IL-1α − 889 C/T (rs1800587) polymorphism was significantly associated with the risks of SSNHL and Ménière disease, whereas IL-1β − 511 C/T polymorphism did not have a significant association with these conditions [6]. The IL-1α − 889 C⁄T polymorphism is located in the promoter region of IL-1α, and carriers of the minor T allele have been shown to have increased transcriptional activity of the gene [9]. Um et al., in their study of a Korean population, observed a significant difference between individuals with SSNHL and controls in the carriage of both the IL-1β − 511T allele and the IL-1β + 3953T allele. This suggests that the IL-1β − 511 and IL-1β + 3953 loci may play a crucial role in the etiopathogenesis of SSNHL [5]. The apparent discrepancy between both studies might be partly due to a difference in ethnic backgrounds between the two populations. The homozygosity for the IL-1β + 3953T allele was associated with a four-fold increase in the production of IL-1β when compared the homozygosity for the C allele [10]. The mechanism by which the IL-1β gene polymorphisms influence SSNHL is probably related to different levels of IL-1β synthesis, secretion, and activity.

Interleukin 6 (IL-6) can function as an anti-inflammatory cytokine. Hiramatsu et al. observed that the IL-6 − 572 C/G (rs1800796) polymorphism was associated with a risk of SSNHL; therefore, they suggested that inflammation of the inner ear might be involved, because permeability of blood vessels in the inner ear is frequently increased in patients with SSNHL in Japan [21]. The IL-6 − 572 C/G polymorphisms are located in the promoter region. Tian et al. reported that the frequency of the G allele at the IL-6 − 572C/G polymorphism was significantly higher among individuals with SSNHL than among healthy individuals in China. Individuals with this allele had significantly higher plasma fibrinogen and C-reactive protein levels than those without it. The allele is also associated with a higher risk of coronary heart disease and myocardial infarction, both of which are related to a higher expression of IL-6 from peripheral blood mononuclear cells [22]. Cadoni et al. demonstrated that the IL-6 − 174G/C gene polymorphism is significantly associated with the risk of SSNHL, which is consistent with a previous finding on serum levels of IL-6 in SSNHL in Italy [23]. Both the IL-6 − 174G/G polymorphism and elevated IL-6 levels in SSNHL patients could suggest that IL-6 has a role in inner ear involvement through atherosclerotic inflammatory events [23].

Interleukin 4 (IL-4) is crucial in mediating isotype switching to IgE. The IL-4 receptor (IL-4R) gene polymorphism is widely associated with atopy and other inflammatory diseases. Nam et al. reported that the IL-4R polymorphism Q576R (G1902A, rs1801275) was a risk factor for SSNHL in Korean patients with sudden deafness [24]. Conversely, Hiramatsu reported no association between SSNHL risk and IL-4R rs1801275 polymorphism in a Japanese population [21]. Polymorphisms of the gene encoding IL-10, an anti-inflammatory cytokine similar to IL-4, failed to show any association with SSNHL risk [24].

Potentially reversible sensorineural hearing loss (SNHL) can be divided into two subgroups: autoimmune inner ear disease (AIED) and SSNHL. In a previous study, Vambutas and colleagues collected inner ear perilymph samples during cochlear implantation in patients with profound deafness from end-stage AIED and used this perilymph to stimulate autologous peripheral blood monocytes (PBMCs). Compared with perilymph from control cochlear implant patients, perilymph from individuals with AIED induced the expression of IL-1 type 2 decoy receptor in PBMCs [25]. Subsequent studies of PBMCs have revealed increased IL-1β expression in steroid-resistant versus steroid-responsive AIED patients. Furthermore, the glucocorticoid dexamethasone was unable to prevent IL-1β release, but the IL-1 receptor antagonist anakinra effectively did [20]. These observations elucidate possible mechanisms of immunologic damage in AIED and indicate the potential of a novel treatment of anakinra or other IL-1β inhibitors or receptor antagonists [26]. Anti-inflammatory agents such as IL-1R antagonists may have potential for the treatment of SSNHL and merit further investigation.

The major limitation of this study is that it involved participants in Eastern Asia only. Further studies involving different ethnic groups are required to generalize our results. Another limitation was that the information about other inflammation conditions unrelated to the ear were unavailable in the design of this study, so that we could not analyze their associations with SSNHL.

In summary, we observed that the SNPs rs2234650 of *IL1R1* and rs4141134 of *IL1R2* were associated with an increased risk of SSNHL. The risk effect of SSNHL may be through inflammation by a decrease in the presence of membrane-bound *IL1R2* for IL-1 for binding. Further functional analyses of SNP rs2234650 and rs2071008 in the *IL1R* and *IL1R2* gene in SSNHL are warranted.

## Conclusions

The results of this study support the influence of genetic polymorphisms of the *IL1R1* and *IL1R2* genes on the risk of SSNHL in a Taiwanese population. The TT genotype of *IL1R1* rs2234650 and CC + CT genotype of the *IL1R2* rs4141134 may be risk genotypes for SSNHL. By contrast, SNPs rs3917225 of *IL1R1* and rs2071008 of *IL1R2* showed no association with SSNHL.

## Data Availability

Not applicable.
